# Multidomain Interventions to Prevent Cognitive Impairment, Alzheimer’s Disease, and Dementia: From FINGER to World-Wide FINGERS

**DOI:** 10.14283/jpad.2019.41

**Published:** 2019-10-10

**Authors:** A. Rosenberg, F. Mangialasche, T. Ngandu, A. Solomon, Miia Kivipelto

**Affiliations:** 1 Department of Neurology, Institute of Clinical Medicine, University of Eastern Finland, Kuopio, Finland; 2 Division of Clinical Geriatrics, Center for Alzheimer Research, Department of Neurobiology, Care Sciences and Society, Karolinska Institutet, Stockholm, Sweden; 3 Aging Research Center, Center for Alzheimer Research, Department of Neurobiology, Care Sciences and Society, Karolinska Institutet and Stockholm University, Stockholm, Sweden; 4 Public Health Promotion Unit, Finnish Institute for Health and Welfare, Helsinki, Finland; 5 Theme Aging, Karolinska University Hospital, Stockholm, Sweden; 6 Stockholms Sjukhem, Research & Development Unit, Stockholm, Sweden; 7 The Ageing Epidemiology Research Unit, School of Public Health, Imperial College London, London, UK; 8 Institute of Public Health and Clinical Nutrition, University of Eastern Finland, Kuopio, Finland; 9 Division of Clinical Geriatrics, Center for Alzheimer Research, Department of Neurobiology, Care Sciences and Society, Karolinska Institutet, Karolinska Universitetssjukhuset, Karolinska Vägen 37 A, QA32, 171 64 Solna, Sweden

**Keywords:** Alzheimer’s disease, cognitive impairment, dementia, multidomain, prevention, randomized controlled trial

## Abstract

Alzheimer’s disease (AD) and dementia are a global public health priority, and prevention has been highlighted as a pivotal component in managing the dementia epidemic. Modifiable risk factors of dementia and AD include lifestyle-related factors, vascular and metabolic disorders, and psychosocial factors. Randomized controlled clinical trials (RCTs) are needed to clarify whether modifying such factors can prevent or postpone cognitive impairment and dementia in older adults. Given the complex, multifactorial, and heterogeneous nature of late-onset AD and dementia, interventions targeting several risk factors and mechanisms simultaneously may be required for optimal preventive effects. The Finnish Geriatric Intervention Study to Prevent Cognitive Impairment and Disability (FINGER) is the first large, long-term RCT to demonstrate that a multidomain lifestyle-based intervention ameliorating vascular and lifestyle-related risk factors can preserve cognitive functioning and reduce the risk of cognitive decline among older adults at increased risk of dementia. To investigate the multidomain intervention in other populations and diverse cultural and geographical settings, the World-Wide FINGERS (WW-FINGERS) network was recently launched (https://alz.org/wwfingers). Within this network, new FINGER-type trials with shared core methodology, but local culture and context-specific adaptations, will be conducted in several countries. The WW-FINGERS initiative facilitates international collaborations, provides a platform for testing multidomain strategies to prevent cognitive impairment and dementia, and aims at generating high-quality scientific evidence to support public health and clinical decision-making. Furthermore, the WW-FINGERS network can support the implementation of preventive strategies and translation of research findings into practice.

**D**ementia is the main cause of disability among older adults, affecting around 50 million people worldwide (1). Driven by population aging, this number is expected to increase rapidly to over 150 million by 2050, creating a major public health and social challenge (2). Alzheimer’s disease (AD) underlies the majority of dementia cases, often in association with vascular neuropathology. Disease-modifying therapies are not yet available for AD, and despite the recent positive signals in some of the ongoing randomized controlled trials (RCTs) testing anti-amyloid compounds, drug trials have mostly reported disappointing results (3,4). Given the evidence emerging from longitudinal observational studies, indicating that late-life cognitive impairment, AD, and dementia are heterogeneous and multifactorial conditions driven by a combination of genetic, vascular, metabolic, and lifestyle-related factors, the potential of dementia prevention through risk factor modification and management has gained increasing attention.

Findings from observational studies need to be substantiated by RCTs, which are considered the gold standard to verify the effect of an intervention. Results of the earlier, smaller, shorter-term prevention RCTs focusing on individual lifestyle components and risk factors have been mostly modest, and evidence from large, long-term trials is only beginning to emerge (5). Importantly, in light of the current knowledge about the complex and multifactorial etiology of late-onset AD and dementia, targeting several risk factors and mechanisms simultaneously, as well as tailoring interventions to individual risk profiles, may be necessary to obtain optimal preventive effects. So far, three large European multidomain lifestyle-based prevention trials have been completed: the Finnish Geriatric Intervention Study to Prevent Cognitive Impairment and Disability (FINGER) (6), the French Multidomain Alzheimer Preventive Trial (MAPT) (7), and the Dutch Prevention of Dementia by Intensive Vascular Care (PreDIVA) (8). The FINGER trial reported significant beneficial intervention effects on the primary outcome, namely change in global cognitive performance, among older ‘at risk’ adults from the general population (6). Notably, exploratory subgroup analyses of MAPT and PreDIVA also suggested cognitive benefits in subpopulations of participants with increased risk of dementia (7–9). Taken together, these studies indicate that administering multidomain lifestyle-based interventions to older at-risk adults may be feasible and effective. However, to fully understand the potential and impact of multidomain preventive interventions, their efficacy and feasibility needs to be explored in diverse populations and contexts worldwide. The FINGER model is now tested and adapted in several new preventive trials globally, and the World-Wide-FINGERS (WW-FINGERS) network (https://alz.org/wwfingers) was launched to support these joint initiatives aiming to reduce the burden of cognitive impairment and dementia. This article will provide an up-to-date overview of the multidomain intervention concept, lessons learned from the recent multidomain RCTs, and future directions in the field.

## Modifiable risk and protective factors of cognitive impairment, Alzheimer’s disease, and dementia: a window of opportunity for prevention

Increasing evidence from long-term prospective cohort studies linking several modifiable risk and protective factors with late-onset dementia and AD has accumulated during the past decades (10, 11). These include vascular and metabolic risk factors and disorders, lifestyle-related, and psychosocial factors. It is also common that neurodegenerative and vascular pathology co-occur, particularly in older adults with dementia, and mixed dementia has been reported to be the most common type of dementia among individuals older than 80 years (12, 13). With regard to several vascular risk factors, the association with dementia risk is modified by age, and different risk factors may be relevant at different time points in life (14). For example, hypertension, obesity, and hypercholesterolemia in mid-life are risk factors for late-onset dementia and AD (15), but the opposite association has been reported later in life and in studies with shorter follow-up times, possibly reflecting reverse-causality (i.e., those factors decrease in the early, asymptomatic stages of dementia most likely as a consequence of the disease) (16, 17). In addition to vascular and metabolic factors (high blood pressure and cholesterol, obesity, diabetes, impaired glucose metabolism), smoking (18), excessive use of alcohol (19), depression (20), as well as other psychosocial factors, such as work-related stress, feelings of hopelessness or loneliness, and infrequent social contacts (21–23), are associated with an increased dementia risk. Protective factors for cognitive impairment, dementia and AD include regular physical activity (24), having a higher formal education (25) and an intellectually demanding and stimulating work (occupational complexity) (26), as well as engaging in cognitively and mentally stimulating leisure activities (27). Social engagement and having a rich social network have also been associated with a reduced risk of dementia and AD (28, 29).

Among pharmacological treatments, both observational studies and RCTs have indicated that antihypertensive drugs may be associated with a reduced risk of AD and dementia (30). The recent large, long-term Systolic Blood Pressure Intervention Trial (SPRINT) Memory and Cognition IN Decreased Hypertension (MIND) RCT reported that intensive blood pressure control (goal <120 mmHg) can be more effective in reducing the risk of cognitive impairment than standard blood pressure control (goal <140 mmHg), although the question of the optimal therapeutic target for systolic blood pressure among oldest old individuals (85+ years) still remains (31). Findings regarding other medications, such as statins, hormone replacement therapy, and non-steroidal anti-inflammatory drugs, are conflicting, as the beneficial effects suggested by observational studies have not been confirmed in RCTs (10).

In relation to diet, some individual nutrients, including omega-3 polyunsaturated fatty acids, vitamins B6 and B12, folate, vitamin D, and vitamins A, C, E (antioxidants), have been associated with a reduced risk of dementia in observational studies (32), although no conclusive evidence has so far emerged from trials testing nutraceutical supplements. Furthermore, regular intake of fish, fruits, vegetables, and nuts have been linked with a reduced risk of cognitive impairment and dementia. Among dietary patterns, cognitive benefits have been reported for different diets which are based on frequent consumption of fruits and vegetables, unsaturated fats, whole grain products, and fish: the Mediterranean Diet, the DASH (Dietary Approaches to Stop Hypertension), the hybrid MIND (Mediterranean-DASH Intervention for Neurodegenerative Delay) diet, and the healthy Nordic diet (33–37). As opposed to single nutrients, the role of healthy and balanced dietary patterns may be more relevant, because nutrients have cumulative and synergistic effects.

Overall, it has been estimated that approximately 35% of dementia cases worldwide could be attributable to nine modifiable risk factors: low educational attainment in early life, midlife hypertension and obesity, diabetes mellitus, smoking, physical inactivity, depression, social isolation and hearing loss over the entire adult life course (38). This indicates clearly a prevention potential across the lifespan. In line with these findings, secular trend studies have indicated that the age-specific incidence and prevalence of dementia may have declined in some Western countries (39), potentially as a result of improved treatment of cardiovascular disease and vascular risk factors, reduction in smoking, increased educational attainment, and an overall improvement in lifestyle. However, the prevalence of dementia has been shown to increase faster than expected in countries like China and Japan (40,41), and with increasing prevalence of some risk factors, such as obesity and type 2 diabetes (42), there is a great need for global efforts to manage risk factors and reduce the burden of dementia.

A key issue to consider in preventive interventions is the fact that multiple risk and protective factors for dementia and AD usually co-occur and interact across the lifespan to determine the individual’s overall risk of dementia. For instance, in the context of interactions between genetic and environmental factors, it has been reported that the harmful effects of unhealthy lifestyle (i.e., unhealthy diet, alcohol misuse, smoking, physical inactivity) may be more pronounced among carriers of apolipoprotein E (*APOE*) ε4 allele, which is the most well-known genetic risk factor of late-onset AD (43). Furthermore, vascular factors can have additive effects (10). Overall, co-occurrence of risk factors, as well as their time- and age-dependent effects, underline the complexity of dementia prevention and imply that a “one-size-fits-all” preventive approach might not be effective. Instead, a tailored, life-course approach targeting multiple risk factors is likely needed for effective prevention of cognitive impairment and dementia. This means that middle-aged and older adults, as well as individuals with heterogeneous risk profiles, may benefit from somewhat different multidomain preventive strategies in order to change their risk profiles.

## From observational studies to clinical trials: large multidomain lifestyle-based interventions

Three pioneering, large, long-term multidomain lifestyle prevention trials have been recently conducted in Europe: the Finnish FINGER trial; the French MAPT trial, and the Dutch PreDIVA trial.

The two-year FINGER trial (NCT01041989) is the first large, long-term, multicenter RCT showing a significant effect of the multidomain lifestyle intervention against cognitive decline among older adults who had increased risk of dementia (6, 44). The FINGER trial enrolled 1260 older adults aged 60–77 years, recruited from previous population-based surveys. Inclusion criteria were as follows: increased risk of dementia based on the CAIDE (Cardiovascular Risk Factors, Aging and Dementia) Dementia Risk Score (≥6 points) (45); and cognitive performance at the mean level or slightly lower than expected for age. Participants were randomized into the multidomain intervention or control group. The multidomain intervention was delivered by trained professionals through both individual sessions and group activities, and it consisted of dietary counseling, exercise, cognitive training, social activities, and monitoring and management of vascular and metabolic risk factors. The control group was offered regular health advice.

The primary outcome of the trial was change in cognitive performance measured by a neuropsychological test battery (NTB) composite score, and secondary cognitive outcomes included domain-specific NTB scores. After two years, the intervention showed significant beneficial effects on the NTB composite score (25% more improvement compared to control), as well as on executive functioning (83% more improvement), processing speed (150% more improvement), and complex memory tasks (40% more improvement). Furthermore, the intervention group had a lower risk of cognitive decline. Follow-ups at 5 and 7 years have been recently completed to determine long-term effects (data analysis is ongoing). The multidomain intervention was safe and well accepted, with high adherence and a low drop-out rate (12%), supporting the feasibility of lifestyle interventions in older at-risk adults. Importantly, the intervention benefits were not limited to cognition: additional favorable effects included body mass index (BMI) reduction (6), improved adherence to dietary guidelines and recommendations (46), and increase in physical activity (6) and health-related quality of life (47). The intervention also improved physical performance and supported daily functioning (48) and lowered the risk of multimorbidity as well as risk of developing new chronic diseases (49). Notably, pre-specified subgroup analyses indicated that the intervention was beneficial regardless of age, sex, education, vascular risk profile and baseline cognitive performance, indicating that the beneficial effects were not limited to a subset of participants, but findings may be generalized to a large population of older adults at increased risk of dementia (50). *APOE* ε4 carriers got clear benefit from the intervention (51).

The three-year MAPT trial (NCT00672685) is a large, long-term RCT combining lifestyle-based intervention with a nutraceutical compound (7). MAPT enrolled 1680 community dwellers aged 70 years or older who had either subjective memory complaints, limitation in one instrumental activity of daily living, or slow gait speed. In the four parallel arms of the RCT, two intervention groups received a multidomain lifestyle intervention consisting of cognitive training and counseling on nutrition and physical activity, either alone or in combination with omega-3 fatty acid supplementation. One intervention group received only the omega-3 fatty acid supplementation, and one arm was assigned to placebo. The primary outcome was change in a cognitive composite score, and secondary outcomes included the individual components of the composite score, other cognitive test scores (e.g. Mini-Mental State Examination MMSE), and the Short Physical Performance Battery and Alzheimer’s Disease Cooperative Study-Activities of Daily Living (ADCS-ADL) Prevention Instrument scores. Although the trial failed to meet its primary outcome, beneficial intervention effects were observed when both groups receiving the multidomain lifestyle intervention were combined. Also, the combined multidomain lifestyle plus omega-3 fatty acid intervention had beneficial effects on some secondary outcomes (ten MMSE orientation items). Moreover, exploratory analyses indicated beneficial effects in specific subgroups of at-risk participants: those with brain amyloid pathology or a CAIDE risk score of ≥6 points (7, 9), which was the same cut-off used in FINGER to select participants.

The PreDIVA (ISRCTN29711771) is a six-year study targeting 3526 older adults aged 70–78 years, recruited via general practices (8). Compared to the FINGER and MAPT participants, the PreDIVA population was rather unselected. The intervention group received a nurse-led multidomain intervention consisting of advice concerning healthy lifestyle and intensive vascular care and risk factor management, including initiation or optimization of antithrombotics and pharmacological treatments for hypertension, dyslipidemia, or diabetes, when necessary. The control group was offered regular care. The main results of the trial did not show any difference in dementia incidence, which was the primary outcome, between the intervention and control groups. However, in the exploratory analyses, a reduction in the incidence of dementia was observed among individuals with untreated hypertension who adhered to the treatment during the trial.

Several important lessons can be learned from these large multidomain prevention trials. First, selecting the right target population at the right time is crucial. Targeting at-risk individuals (as opposed to an unselected population) is likely the most feasible strategy. Second, the FINGER trial demonstrated the importance of starting early enough: the prevention potential of a multidomain lifestyle intervention, especially if not combined with pharmacological treatments, may be highest among relatively healthy and younger old adults. Finally, the content of the intervention is crucial. The intervention may need to be intensive enough and preferably include also active counseling and coaching delivered in different ways (not only advice). Based on the content and duration of the intervention sessions and study visits, the FINGER intervention was the most intensive, and the participants attended both group and individual sessions. Despite the relatively intensive nature of the intervention, adherence was high, as approximately 72% of the participants reported at least some engagement in all intervention components. Thus, the FINGER multidomain intervention seemed feasible, pragmatic, and not too strenuous. Designing and adapting the content of the intervention for various target populations is essential to optimize the effect. Finally, the choice of an appropriate outcome measure to assess intervention effects is also important. Incidence of dementia is a robust outcome, and trials with such outcome would require a large sample size and long-term follow-up, especially when targeting cognitively healthy older adults. For this population, there is currently no gold standard measure to detect cognitive changes predictive of future dementia. However, composite cognitive scores capturing several cognitive domains may be useful (52), not only to detect early changes typical for AD, but also for vascular cognitive impairment, since both disorders often co-occur in advanced age.

## Other innovative multidomain preventive strategies

Building upon the experiences of the preventive RCTs conducted so far, the next generation of multidomain prevention trials has started to incorporate and utilize novel technologies and tools, such as eHealth and mHealth, to optimize the delivery of multidomain interventions. One example of an Internet-based eHealth study is the Healthy Aging Through Internet Counselling in the Elderly (HATICE, ISRCTN48151589), which is a European 18-month RCT testing the efficacy of an Internet platform in improving self-management of cardiovascular risk factors for prevention of cardiovascular disease and cognitive decline (53). The trial enrolled 2724 non-demented, computer literate community-dwellers aged 65+ from Finland, France, and the Netherlands. Participants were required to have at least two cardiovascular risk factors and/or history of cardiovascular disease or diabetes. Participants were randomized 1:1 to intervention and control groups. The intervention group had access to an interactive Internet platform, designed to encourage lifestyle changes with the remote support of a lifestyle coach, according to national and European guidelines for cardiovascular risk factor management (54). The control platform included only basic health information and no interactive features or coach support. The trial has been completed, and data analysis is ongoing. If the delivery of preventive interventions through Internet or e.g. via mobile applications proved to be feasible and effective and induced sustained behavioral changes, it could support self-management and be a cost-effective way to reach and involve a large population across the world.

While the FINGER, MAPT, and PreDIVA trials targeted older adults from the general population, some new multidomain prevention studies focus on at-risk populations in clinical settings. One particularly relevant target population for multidomain prevention are individuals with prodromal AD. For this more advanced and symptomatic state of AD dementia risk, lifestyle and vascular changes alone may not be sufficient. Rather, a combination of lifestyle and pharmacological approaches may be necessary to prevent or delay the onset of dementia. There are currently no proven therapeutic options available for such individuals, but in the multinational European LipiDiDiet trial (NTR1705) (55), the effects of the medical food product Fortasyn Connect (Souvenaid) were investigated in 311 memory clinic patients with prodromal AD, as defined by the International Working Group (IWG)-1 research criteria (56). Fortasyn Connect is a mixture of multiple nutrients, such as vitamins, polyunsaturated fatty acids, and phospholipids, which improves the formation and function of synapses (57). The primary outcome of the LipiDiDiet trial was change in cognitive performance measured with an NTB composite score. Secondary outcomes included change in e.g. memory scores, Clinical Dementia Rating-Sum of Boxes (CDR-SB), and brain volume. The two-year core trial was completed in 2015. Despite no significant effect on the primary outcome, group differences in favor of the treatment group were observed for cognitive and functional outcomes (45% less worsening in the CDR-SB in the intervention group), and hippocampal atrophy (26% less deterioration in the intervention group) (55). Notably, the observed decline in the NTB in the control group was smaller than expected. Analyses of the intervention effects at 36 months will be completed soon.

## Going global: from FINGER to World-Wide FINGERS

Following the encouraging results of the FINGER trial, the World-Wide FINGERS (WW-FINGERS, https://alz.org/wwfingers) network was launched in July 2017 in connection to the Alzheimer’s Association International Conference in London (founder and scientific lead: Professor Miia Kivipelto; hosted by Alzheimer Association). By collectively convening international research teams under the WW-FINGERS leadership of Prof. Miia Kivipelto and Dr. Maria Carrillo, WW-FINGERS will facilitate data sharing and joint analysis across studies, establish opportunities for joint initiatives across country borders, and strengthen the potential evidence-base for multidomain lifestyle interventions.

**Figure 1:**
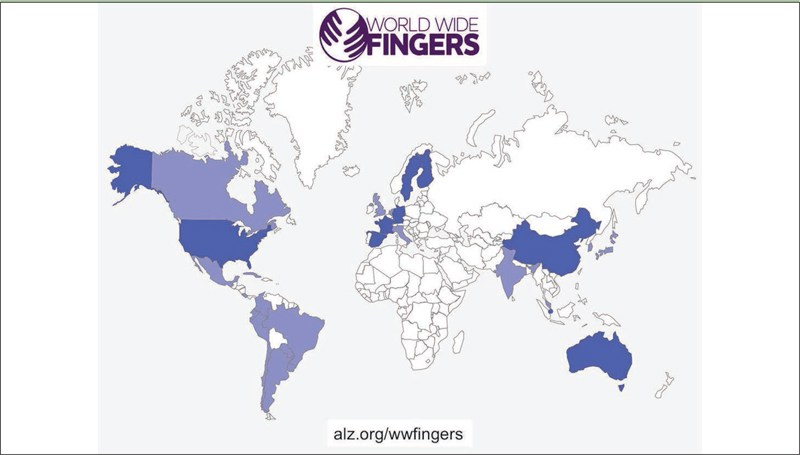
World map with countries which are involved in the WW-FINGERS network. Blue indicates involvement in ongoing WW-FINGERS studies. Studies are currently planned in countries marked with purple

This initiative supports and coordinates other trials worldwide in testing the feasibility and efficacy of FINGER-type preventive interventions in different at-risk populations, across diverse geographical and cultural settings. All WW-FINGERS trials share the same key concept of a pragmatic multidomain approach, i.e. targeting several modifiable risk factors simultaneously. WW-FINGERS will facilitate data sharing and joint analysis across studies, and to ensure comparability of the results and to facilitate pooling of accumulating data, the trials aim to use common core outcome measures. At the same time, local and cultural adaptations will be applied in relation to the content and delivery method of the intervention. For example, dietary counseling will follow national recommendations while taking into account country- or region-specific habits, and pharmacological vascular risk factor management, when applicable, will be based on national care guidelines. This is essential to improve engagement and adherence, and subsequently, to facilitate the effective and sustainable implementation of preventive strategies. Several countries worldwide have joined the WW-FINGERS network and are currently at different stages of planning and conducting their FINGER-type prevention trials (Figure 1). Recruitment is already ongoing in several trials.

## WW-FINGERS trials

The U.S. Study to Protect Brain Health Through Lifestyle Intervention to Reduce Risk (U.S. POINTER), supported by the Alzheimer’s Association, aims to test the FINGER intervention in a more diverse US population. It is a two-year trial targeting 2000 older adults aged 60–79 years with normal cognition but increased risk for future cognitive decline. The trial will compare two lifestyle-based interventions (structured vs self-guided lifestyle intervention), which vary in their intensity and structure. Another ongoing trial testing the FINGER-based model is the randomized controlled Multimodal INtervention to delay Dementia and disability in rural China (MIND-CHINA), aiming at recruiting up to 3500 older adults aged 60–79 years who are living in rural areas of the Shandong province. The MIND-CHINA trial uses cluster randomization by village and includes two intervention arms and a control arm. Due to high prevalence of untreated vascular risk factors in this population, the trial will focus on the management and treatment of these factors. Participants in the vascular intervention group will be provided with pharmacological control and management of three major vascular risk factors (hypertension, dyslipidemia, diabetes); the multidomain intervention group will have both the management of the vascular risk factors and a multidomain lifestyle intervention. In the lifestyle intervention, special emphasis will be placed on reducing salt intake, which is a key dietary challenge in China. In Singapore, the six-month feasibility study SINGapore GERiatric intervention study to reduce physical frailty and cognitive decline (SINGER) targeting 70 participants with mild/moderate frailty and/or cognitive impairment is ongoing. The two-year study AUstralian-Multidomain Approach to Reduce Dementia Risk by PrOtecting Brain Health with Lifestyle intervention (AU-ARROW) is currently planned in Australia. Another Australian multidomain prevention trial, the ongoing three-year Maintain Your Brain (MYB) trial, is associated with WW-FINGERS (study design and outcomes not fully harmonized with other WW-FINGERS studies). The MYB RCT randomized 6236 non-demented community-dwellers aged 55–77 years. Assessments and interventions are conducted online, and the multidomain eHealth intervention consists of exercise, cognitive training, dietary advice, guidance to stop smoking and reduce alcohol consumption, blood pressure and cholesterol management, and cognitive behavior therapy to manage depressive symptoms and to facilitate social interaction.

Another initiative within the WW-FINGERS network is the multinational European collaboration project MIND-AD - Multimodal preventive trials for Alzheimer’s Disease: towards multinational strategies, which is based on the promising results of the FINGER and LipiDiDiet trials. In the ongoing six-month Multimodal Preventive Trial for Alzheimer’s Disease (MIND-ADmini) (NCT03249688) pilot trial (extended six-month follow-up in some countries), a multidomain lifestyle intervention derived from the FINGER trial is tested both alone and in combination with Souvenaid among individuals with prodromal AD and vascular or lifestyle-related risk factors. The control group receives usual care and regular health advice. Trial participants were recruited from Finland, France, Germany, and Sweden, and the main objective is to assess the feasibility of the multidomain intervention in this population. MIND-AD can serve as a model and platform for future trials combining nonpharmacological and pharmacological approaches to prevent or delay the onset of dementia. A master protocol for combination therapy is currently under development. In addition to the abovementioned trials, WW-FINGERS interventions are also planned in several other countries including Japan, Canada, UK, the Netherlands, Spain, Italy, India, South Korea, Malaysia, and several Latin American countries (LATAM FINGER project) (Figure 1).

## Conclusions

Prevention has been recognized as pivotal in halting the expected worldwide increase of AD and dementia cases. Successful preventive approaches should be feasible, accessible, cost-effective, and sustainable for populations in different geographical, economic, and cultural settings. Several modifiable risk factors, which can be managed to promote brain health and reduce the risk of late-life AD and dementia, have been identified. Yet, the majority of the observational studies on risk and protective factors have been conducted in high-income countries, with few findings available from low- and middle-income countries, which are facing the highest rise in dementia prevalence and incidence. In fact, by 2050, 68 % of all people with dementia worldwide are expected to live in low- and middle-income countries (1). The World Health Organization has invited experts worldwide to produce a global action plan and guidelines for cognitive decline and dementia risk reduction (58), and studies documenting prevalence and time-trends of risk factors in low- and middle-income countries can help develop preventive models for these areas.

The multidomain preventive approach has already proven its efficacy in other age-related chronic conditions (diabetes mellitus, cardiovascular disease (59,60)), and can facilitate also the reduction of dementia risk by addressing the multifactorial, complex, and heterogeneous nature of late-life cognitive impairment, AD, and dementia. Importantly, it offers prevention potential on a large scale, with possibilities for worldwide implementation. Country-specific adaptations will be crucial to ensure effective implementation of multidomain preventive interventions in different cultural, geographical, and economical settings, as well as public health care systems. Introduction of innovative eHealth and mHealth tools can facilitate implementation and monitoring of the interventions, while reducing costs and reaching larger regions and populations. The proportion of older adults using Internet is increasing, supporting the use of eHealth-based approaches, but feasibility is a key issue and is actively investigated. For instance, in the HATICE RCT older adults were involved in the development of the Internet platform that was used to deliver the multidomain intervention, in order to optimize its acceptability and use (61). Similar efforts may be needed also in future trials investigating eHealth or mHealth tools.

The complex nature of late-life cognitive impairment, AD, and dementia translates into a need to identify different risk profiles in order to develop tailored preventive strategies, within the framework of preventive precision medicine. WW-FINGERS is a landmark initiative, which will facilitate identification of efficacious preventive approaches for specific risk profiles and cost-effective implementation of such approaches in different settings. The WW-FINGERS model can be further developed to integrate pharmacological treatments, as the AD drug development field advances and succeeds in identifying effective disease-modifying compounds. Multidomain schemes combining pharmacological and non-pharmacological interventions can be developed and tested to define secondary and tertiary preventive strategies across the full spectrum of AD.

The WW-FINGERS network facilitates international collaboration in dementia prevention and provides an opportunity to harmonize prevention studies, as well as share experiences and data to obtain maximum scientific impact. Furthermore, the network aims at generating high-quality scientific evidence to support public health and clinical decision-making. This global joint effort can also have a key role in promoting rapid and effective dissemination and implementation of research findings.

## Notes

*Ethical standards:* All studies presented in this article are conducted according to the principles of the Declaration of Helsinki and following the guidelines for Good Clinical Practice. Studies are approved by local ethics committees and all participants provided written informed consent.
